# Unraveling Protracted Neuropsychiatric Symptoms in a Patient With Altered Post‐Bariatric Pharmacokinetics: A Diagnostic Puzzle

**DOI:** 10.1155/crps/8340699

**Published:** 2026-06-15

**Authors:** Akbar Ali, Christopher E. Potts, Dakota May

**Affiliations:** ^1^ Joan C. Edwards School of Medicine, Marshall University, Huntington, WV, USA, marshall.edu

## Abstract

**Objective:**

To report a complex case of serotonin toxicity that evolved into a persistent neuropsychiatric syndrome, complicating the differential diagnosis between protracted toxicity, prolonged SSRI discontinuation syndrome, acute hepatic porphyria, and functional neurological disorder (FND).

**Method:**

We present the case of a 61‐year‐old male with a history of gastric sleeve surgery and major depressive disorder (MDD) who developed acute serotonin syndrome (SS) following the concomitant use of fluoxetine, buspirone, and linezolid.

**Results:**

While acute autonomic instability resolved upon cessation of serotonergic agents, the patient developed subacute, evolving neurological deficits. Workup revealed an *SLC6A4* polymorphism and elevated urinary porphyrins, initially prompting Hemin therapy, though genetic testing for porphyria was negative. Tertiary evaluation at the Cleveland Clinic ultimately confirmed a diagnosis of FND, psychogenic non‐epileptic seizures (PNES), and chronic migraine. Management remains challenging due to medication sensitivity, including recent parasomnias during a cautious trial of vilazodone.

**Conclusions:**

This case highlights the diagnostic complexity following SS in patients with altered gastrointestinal anatomy and genetic variances. It emphasizes how an acute organic insult—whether serotonin toxicity, prolonged SSRI discontinuation, or a combination of both—can serve as a physiological trigger for complex FND and PNES, requiring multidisciplinary management. The limitations inherent in single‐case observations are discussed.

## 1. Introduction

Serotonin syndrome (SS) is typically characterized as an acute, self‐limiting drug reaction that resolves within 24–72 h of medication discontinuation. However, the trajectory of recovery can be obscured by complicating factors such as altered drug metabolism, SSRI discontinuation phenomena, and psychological sequelae. Patients with a history of bariatric surgery represent a unique pharmacokinetic population where absorption and clearance of psychotropics may be unpredictable.

We present a case where “classic” SS evolved into a months‐long diagnostic dilemma. This report dissects the intersection of prolonged half‐life serotonergics, genetic transporter findings (*SLC6A4*), incidental metabolic findings (porphyrinuria), and the emergence of functional neurological disorder (FND) triggered by acute physiological stress. Importantly, we acknowledge alternative explanations—including prolonged fluoxetine discontinuation syndrome and psychiatric relapse—and discuss the limitations of the causal inferences that can be drawn from a single case.

## 2. Case Report

### 2.1. History of Present Illness

Mr. A, a 61‐year‐old male, presented to the psychiatric clinic for management of severe panic attacks and persistent neurological symptoms. His medical history included a gastric sleeve procedure, major depressive disorder (MDD), and posttraumatic stress disorder (PTSD).

Four months prior to presentation, he was treated for a urinary tract infection with linezolid, a reversible monoamine oxidase inhibitor (MAOI). Concurrently, he remained on fluoxetine (40 mg) and buspirone (30 mg), despite recommendations to wash out the SSRI. Within 48 h, he developed acute confusion, diaphoresis, tremor, and hyperreflexia. He met the Hunter Serotonin Toxicity Criteria (clonus, agitation, diaphoresis, hypertonia, and hyperthermia).

### 2.2. Clinical Evolution and Timeline

Following the discontinuation of all serotonergic agents, the patient’s acute autonomic instability (tachycardia and hyperthermia) resolved within days. However, he did not return to his neurological baseline. Over the ensuing months, his clinical picture shifted from toxic encephalopathy to a complex constellation of episodic deficits (Table 1).

**Table 1:** Evolution of clinical symptoms and diagnostic markers.

Phase	Timeframe	Clinical features	Key interventions/findings
I. Acute toxicity	Week 0	Confusion, agitation, inducible clonus, diaphoresis, fever (>38°C)	Diagnosis: serotonin syndrome Action: discontinued fluoxetine, buspirone, linezolid
II. Subacute persistence	Weeks 1–3	Resolution of fever/autonomic instability. Persistence of “internal tremors,” severe anxiety, and “brain zaps“	Observation: symptoms attributed to fluoxetine washout (long half‐life) and possible SSRI discontinuation syndrome
III. Neuro‐psychiatric evolution	Months 2–3	Emergence of episodic right‐sided facial droop (see Investigations), slurred speech, wide‐based gait, and “electric shock” sensations	Workup: MRI brain (unremarkable). Lumbar puncture not performed (see Investigations) Labs: elevated urinary porphyrins. LFTs mildly elevated; albumin low‐normal; renal function and infection markers within normal limits at time of testing (see Discussion)
IV. Therapeutic trial	Month 4	Continued episodic weakness and panic	Hematology: trial of Hemin infusions Result: subjective transient improvement, but genetics negative
V. Tertiary diagnosis	Month 5	Worsening depression (PHQ‐9:25) and anxiety (GAD‐7:21). Onset of “flashes” and intrusive thoughts	Diagnosis: FND, PNES, migraine Action: referred to FND clinic (PT/OT/SLP). Started vilazodone 5–10 mg
VI. Current status	Month 6+	Complex parasomnias (sleepwalking), tinnitus, and continued tremors	Plan: evaluating vilazodone vs. diazepam contribution to parasomnias

### 2.3. Investigations

• Neuroimaging: MRI Brain demonstrated no acute intracranial pathology, restricted diffusion, or structural lesions to explain the facial droop. It should be noted that up to 20% of cases may be missed on a single MRI, and the diagnostic yield of neuroimaging is influenced by the timing of the scan, particularly in the context of autoimmune encephalitis [1].
• Lumbar puncture: a lumbar puncture with cerebrospinal fluid (CSF) analysis—including PCR for infectious etiologies and antibody screening for autoimmune encephalitis—was not performed during this patient’s workup. This represents a limitation in the neurological assessment, as CSF analysis could have further excluded infectious or autoimmune contributions to the patient’s altered mental status, particularly given the context of an active urinary tract infection at onset. The decision to defer lumbar puncture was made by the treating team based on the clinical trajectory and the absence of progressive encephalopathic features at the time of neurological evaluation; however, the authors acknowledge that this investigation would have strengthened the diagnostic workup.
• Characterization of facial droop: the right‐sided facial droop was documented on clinical examination and was not solely patient‐reported. Importantly, the droop was episodic and fluctuating in nature: it was observed to vary in severity across visits, was not present continuously, and did not follow a fixed upper or lower motor neuron distribution. On detailed examination, the facial asymmetry was noted to diminish when the patient was distracted and to intensify during periods of emotional distress and focused clinical observation—features more consistent with a functional etiology than with cerebrovascular compromise or a structural lesion [2]. The absence of corresponding findings on MRI further supports this interpretation.
• Pharmacogenomics (GeneSight)
• SLC6A4: homozygous for the short promoter polymorphism, associated with reduced serotonin transporter expression.
• MTHFR: heterozygous for C677T polymorphism.
• ADRA2A: homozygous C allele (moderately reduced response).
• Porphyria screen: urine analysis revealed elevated coproporphyrin I, heptacarboxyl, pentacarboxyl, and uroporphyrins. Subsequent genetic testing for acute intermittent porphyria (AIP) and other porphyrias was negative.
• Metabolic and hepatic assessment: at the time of the porphyria workup, liver function tests (AST and ALT) were mildly elevated, albumin was low‐normal, and renal function (BUN and creatinine) and inflammatory markers (CRP, CBC with differential) were within normal limits. These findings are discussed further in the context of the elevated urinary porphyrins below.

### 2.4. Therapeutic Interventions

Phase I management consisted of immediate discontinuation of all serotonergic agents (fluoxetine, buspirone, and linezolid) with supportive care for the acute autonomic instability. During the subacute phase (Weeks 1–3), no specific pharmacological intervention was initiated, as residual symptoms were attributed to fluoxetine washout and possible SSRI discontinuation syndrome. In Month 4, a trial of Hemin infusions was undertaken by hematology following identification of elevated urinary porphyrins, yielding a subjective but inconsistent response. Following tertiary evaluation confirming FND, psychogenic non‐epileptic seizures (PNES), and chronic migraine (Month 5), the patient was referred to a multidisciplinary FND clinic encompassing physical therapy, occupational therapy, and speech–language pathology. Pharmacological reintroduction of antidepressant therapy was approached with caution given the patient’s demonstrated serotonergic sensitivity, with vilazodone titrated from 5 to 10 mg.

### 2.5. Follow‐Up and Outcomes

At the time of this report (Month 6+), the patient continues to experience complex parasomnias (sleepwalking), tinnitus, and persistent tremors. Psychiatric symptom burden at Month 5 was severe, with a PHQ‐9 score of 25 and a GAD‐7 score of 21. The central management challenge remains balancing adequate psychiatric pharmacotherapy against the patient’s marked serotonergic sensitivity. The relative contributions of vilazodone and concurrent low‐dose diazepam to the observed parasomnias remain under evaluation. The patient is actively engaged with the multidisciplinary FND clinic and continues follow‐up with neurology and psychiatry.

### 2.6. Patient Perspective

Mr. A described the period following the acute episode as profoundly disorienting. He reported feeling that “something had switched off” in his brain after beginning the antibiotic course, and expressed frustration with the extended diagnostic uncertainty, stating that not knowing what was wrong with him was, at times, as distressing as the symptoms themselves. He described the episodic neurological deficits—particularly the facial weakness and gait disturbance—as unpredictable and socially isolating. He noted that receiving the FND diagnosis at tertiary evaluation provided a degree of relief in that it offered an explanatory framework for his experience, though he acknowledged that the path to recovery remained uncertain. He expressed appreciation for the multidisciplinary approach to his care and remains engaged with treatment.

## 3. Discussion

Based on the totality of clinical and investigative evidence—including specialist evaluation at a tertiary FND center—the most likely diagnosis accounting for the chronic neurological phase of this case is FND with PNES. This conclusion is supported by the positive semiology of functional weakness on clinical examination, the absence of structural or autoimmune pathology on neuroimaging, and the temporally plausible relationship with an acute physiological precipitant in the form of SS [3]. It is acknowledged that, in a single‐case report, causal inferences remain inherently speculative, and alternative diagnoses cannot be fully excluded on clinical grounds alone. The competing diagnoses of protracted serotonin toxicity, prolonged SSRI discontinuation syndrome, and acute hepatic porphyria are considered less likely as primary drivers of the chronic syndrome and are addressed in detail below.

The differential diagnosis for this patient’s persistent symptoms centers on three competing etiologies: protracted serotonin toxicity (or prolonged SSRI discontinuation syndrome), seronegative porphyria, and FND (Table 2).

**Table 2:** Comparison of differential diagnoses.

Feature	Protracted SS/SSRI discontinuation	Acute hepatic porphyria	FND/PNES
Autonomic abnormalities	Early only	Possible	Usually absent
Focal deficits	Uncommon	Possible neuropathy	Common/inconsistent
Lab findings	None specific	Elevated PBG/ALA/porphyrins	Normal
Response to Hemin	Unclear	Expected	Placebo response possible
Timeline	Typically days–weeks	Episodic	Variable

### 3.1. Differential Diagnosis

1. Protracted SS vs. prolonged SSRI discontinuation and psychiatric relapse

While SS is typically acute, the pharmacokinetic context of this patient raises the possibility of a protracted course. Fluoxetine’s active metabolite, norfluoxetine, has a half‐life of 7–15 days, which can be further prolonged in specific metabolic contexts.

• Bariatric pharmacokinetics: gastric sleeve surgery alters gastric emptying and pH, which can lead to erratic absorption and potentially higher peak serum concentrations of lipophilic drugs like SSRIs [4].
• Genetic finding: the patient’s *SLC6A4* short‐promoter genotype results in reduced serotonin transporter expression. It is worth noting, however, that the clinical significance of *SLC6A4* polymorphisms in the context of SS remains speculative. While the short‐promoter variant has been associated with altered serotonergic signaling in affective disorders [5], its role in modulating the duration or severity of serotonin toxicity has not been established in controlled studies. We present this finding as hypothesis‐generating rather than as a confirmed mechanistic contributor, and acknowledge the risk of overinterpreting pharmacogenomic data in a single‐case context.

An important alternative interpretation is that the subacute symptoms in Phase II—“brain zaps,” internal tremors, anxiety—may represent a prolonged SSRI discontinuation syndrome rather than residual serotonin toxicity per se. Fluoxetine discontinuation syndrome is generally considered uncommon due to the drug’s long half‐life; however, in a patient with altered gastrointestinal absorption post‐bariatric surgery, the pharmacokinetic profile may deviate from population norms, making withdrawal effects more unpredictable. Furthermore, the chronic‐phase symptoms (worsening depression, panic, intrusive thoughts) may partially reflect psychiatric relapse in the setting of abrupt cessation of antidepressant therapy, compounded by the psychological trauma of the acute serotonin toxicity event. Disentangling protracted toxicity from discontinuation phenomena and psychiatric relapse is not possible with certainty on clinical grounds alone, and we acknowledge this ambiguity as a core limitation of this report.

2. The porphyria confounder

The elevation of urinary porphyrins introduced significant diagnostic noise. While elevated porphobilinogen (PBG) is the hallmark of acute hepatic porphyrias, nonspecific porphyrinuria (elevation of coproporphyrins/uroporphyrins) can occur in the setting of hepatic dysfunction, malnutrition, or severe physiological stress [6, 7].

In this patient, the most likely explanation for the elevated urinary porphyrins is secondary (nongenetic) porphyrinuria related to a combination of factors present at the time of testing: mildly elevated liver transaminases suggesting hepatic stress, low‐normal albumin potentially reflecting suboptimal nutritional status in the context of prior bariatric surgery, and the physiological stress of the acute serotonin toxicity episode itself. Renal function and infection markers were normal at the time of porphyria workup, making renal impairment and active systemic infection unlikely contributors to an encephalopathic syndrome or to the porphyrin elevations. The pattern of porphyrin elevation—predominantly coproporphyrins and uroporphyrins without significant PBG or ALA elevation—is characteristic of secondary porphyrinuria rather than a primary enzymatic defect [6].

The patient’s subjective response to Hemin was likely a placebo effect or coincidental to the natural waxing and waning of symptoms, supported by the lack of a reproducible clinical response across infusions and by the negative confirmatory genetics. This highlights the danger of relying solely on urinary markers without genetic confirmation and without adequately accounting for secondary causes.

3. FND and PNES

Tertiary evaluation confirmed FND and PNES as the primary drivers of the chronic phase. FND is frequently precipitated by a “physical trigger” [3]; it is speculated that, in this case, the acute physiological stress of the SS episode may plausibly have served in this role, though a direct causal relationship cannot be confirmed. The discordance between the severity of the neurological complaints (unable to walk, slurred speech) and the normal MRI/neuro‐exam findings supports this. As noted above, the episodic facial droop demonstrated features characteristic of functional weakness—namely, fluctuation with distraction, intensification under emotional distress and direct observation, and absence of a fixed cranial nerve distribution—rather than findings suggestive of cerebrovascular compromise [2].

The patient’s subsequent decompensation (PHQ‐9:25) and emergence of parasomnias during a cautious rechallenge with vilazodone (a SPARI) highlights the difficulty of treating the psychiatric sequelae in a sensitized nervous system. The “electric shocks” and “flashes” likely represent a functional overlay on top of genuine sensory sensitization from the initial serotonin toxicity, though it is not possible to confirm this with certainty.

## 4. Limitations

Several limitations of this case report warrant explicit acknowledgment. These limitations are organized into two domains diagnostic constraints and data or investigational constraints.

### 4.1. Diagnostic Constraints

First, as a single‐case observation, causal inferences must be drawn with caution. The temporal sequence of events is suggestive but does not establish causality between the serotonin toxicity episode, the pharmacogenomic findings, and the subsequent development of FND/PNES. Second, the distinction between protracted SS, prolonged SSRI discontinuation syndrome, and psychiatric relapse cannot be made definitively in this case, as no serum serotonin or norfluoxetine levels were obtained during the subacute phase. Third, a lumbar puncture was not performed, leaving infectious and autoimmune encephalitis incompletely excluded, although the overall clinical trajectory and tertiary‐center evaluation support the final diagnosis of FND.

### 4.2. Data and Investigational Constraints

Fourth, the clinical significance of the *SLC6A4* short‐promoter polymorphism in this context is speculative, as controlled evidence linking this genotype to the duration or severity of SS is lacking. Fifth, the elevated urinary porphyrins, while most likely secondary, were not followed longitudinally to confirm resolution, which would have further supported their classification as incidental. Finally, the reliance on a commercial pharmacogenomic panel (GeneSight) introduces the limitation that such panels have variable clinical validation, and their findings should be interpreted within the broader clinical context rather than as standalone diagnostic evidence.

## 5. Conclusion

This case serves as a reminder that the clinical aftermath of SS is not always linear, particularly in post‐bariatric patients with altered pharmacokinetics. The interplay among acute serotonin toxicity, possible SSRI discontinuation phenomena, incidental metabolic findings, and underlying psychiatric vulnerability can create a diagnostic picture of considerable complexity. Furthermore, this case illustrates how an acute organic insult can serve as the “trigger event” for a complex FND and PNES, requiring a multidisciplinary approach (PT, OT, and psychology) rather than purely pharmacologic management. Clinicians should be aware of the limitations of pharmacogenomic and urinary porphyrin data in isolation and should pursue a comprehensive workup—including CSF analysis when clinically indicated—before anchoring on a single diagnosis.

## Funding

The authors received no specific grant or financial support from any public, commercial, or not‐for‐profit funding agency for the preparation of this case report.

## Disclosure

All identifying details have been removed, and the authors have confirmed that the details of the case have been appropriately disguised to protect patient privacy.

## Consent

Written informed consent was obtained from the patient for publication of this case report and any accompanying images.

## Conflicts of Interest

The authors declare no conflicts of interest.

## Data Availability

Research data are not shared.
